# Tranexamic acid in non-traumatic intracranial bleeding: a systematic review and meta-analysis

**DOI:** 10.1038/s41598-021-94727-y

**Published:** 2021-07-27

**Authors:** Jean-Baptiste Bouillon-Minois, Carolyne Croizier, Julien S. Baker, Bruno Pereira, Farès Moustafa, Justin Outrey, Jeannot Schmidt, Nicolas Peschanski, Frédéric Dutheil

**Affiliations:** 1 CNRS, LaPSCo, Physiological and Psychosocial Stress, CHU Clermont–Ferrand, Emergency Medicine, Université Clermont Auvergne, 63000 Clermont–Ferrand, France; 2 Department of Hematology and Cell Therapy, CHU Clermont–Ferrand, 63000 Clermont–Ferrand, France; 3 Centre for Health and Exercise Science Research, Department of Sport, Physical Education and Health, Hong Kong Baptist University, Kowloon Tong, Hong Kong; 4 Clinical Research and Innovation Direction, CHU Clermont-Ferrand, 63000 Clermont-Ferrand, France; 5 Emergency Department, CHU Clermont-Ferrand, 63000 Clermont-Ferrand, France; 6 Emergency Department, CHU de Besançon, Besançon, France; 7 Emergency Department & SAMU, University of Rennes Hospital, 35000 Rennes, France; 8 Rennes-1 University School of Medicine, 35000 Rennes, France; 9 CNRS, LaPSCo, Physiological and Psychosocial Stress, CHU Clermont–Ferrand, Occupational and Environmental Medicine, Université Clermont Auvergne, WittyFit, 63000 Clermont–Ferrand, France; 10 Emergency Department, CHU Clermont-Ferrand, 58, Rue Montalembert, 63000 Clermont-Ferrand, France

**Keywords:** Therapeutics, Neurological disorders

## Abstract

Non-traumatic intracranial bleeding (NTIB), comprising subarachnoid hemorrhage (SAH) and intra-cranial bleeding (ICH) is a significant public health concern. Tranexamic acid (TXA) is a promising treatment with benefits yet to be fully demonstrated. We conducted a systematic review and meta-analysis on the impact of TXA on mortality in NTIB. We searched the PubMed, Cochrane Library, Google Scholar and ScienceDirect databases for studies reporting mortality data following the use of TXA in NTIB for comparisons with a control group. We computed random-effect meta-analysis on estimates of risk and sensitivity analyses. We computed meta-regression to examine the putative effects of the severity of NTIB, sociodemographic data (age, sex), and publication date. Among potentially 10,008 articles, we included 15 studies representing a total of 4883 patients: 2455 receiving TXA and 2428 controls; 1110 died (23%) during the follow-up. The meta-analysis demonstrated a potential of 22% decrease in mortality for patients treated by TXA (RR = 0.78, 95%CI 0.58–0.98, p = 0.002). Meta-regression did not demonstrate any influence of the severity of NTIB, age, sex, length of treatment or date of publication. Sensitivity analyses confirmed benefits of TXA on mortality. TXA appears to be a therapeutic option to reduce non-traumatic intracranial bleeding mortality, particularly in patients with SAH.

## Introduction

Spontaneous—non-traumatic—intracranial bleeding (NTIB) is a significant public health concern causing close to three million deaths globally in 2017—an increase of 12.5% compared to 2007 and is similar to ischemic stroke mortality^1^. NTIB comprises subarachnoid hemorrhage (SAH)—whose primary source is the rupture of an intracranial aneurysm—and intracerebral hemorrhage (ICH). Over the past four decades, the development of intensive care support, control of blood pressure, and early diagnosis-exclusion of aneurysm induced a decrease in mortality in populations under 50 years old. During the first part of the twenty-first century, the development of endovascular coiling care^2^ offered hope to provide better care for all patients. However, this development will probably take decades to emerge worldwide. Contrary to ischemic strokes, whose prognostic was improved with the generalization of thrombolytic therapy^3^, no drug has been developed to significantly decrease mortality among patients with ICH. Survivors of SAH or ICH both suffer from long-term disabilities such as cognitive impairments, low working abilities, fewer social activities, and poor quality of life^4^.

Tranexamic acid (TXA) is a lysine analog fixing on plasminogen to inhibit fibrinolysis and reduce bleeding. The drug was discovered in the 1960s and first used in the 70s on post-partum hemorrhage and has also been used in orthopedic surgery. Its intravenous bioavailability is close to 100%, with an immediate peak plasma concentration. In the 70s and more recently, in 2010, researchers were interested in the impact of TXA on patients suffering from NTIB. After the 2011 CRASH-2 trial^5^, TXA has been classified in the WHO model list of essential medicines for bleeding trauma. However, only a limited number of studies have provided statistical evidence for the benefits of tranexamic acid on mortality among patients suffering from non-traumatic intracranial bleeding.

As a result, we decided to conduct a systematic review and meta-analysis on the impact of TXA on mortality in NTIB patients.

## Methods

### Search strategy and selection criteria

We conducted a systematic search on PubMed, Cochrane Library, Google Scholar, and ScienceDirect to identify all prospective studies that reported a comparison of tranexamic acid versus placebo injection or control care. The research was not limited to specific years, and no language restriction was applied. The following relevant MESH terms/keywords were used until January 27th, 2021: ("Tranexamic Acid" OR "TXA") AND ("subarachnoid hemorrhage" OR "SAH" OR "intracerebral hemorrhage" OR "ICH"). Reference lists of all publications meeting the inclusion criteria were manually searched to identify any further studies that were not found using the electronic search, as well as reference lists from reviews. Three authors (JBBM, CC, and NP) conducted the same research strategy separately. Databases, titles, and abstracts were screened to decide which articles could be included. Two other authors (FD and JS) were asked to review the articles when the first researchers disagreed on suitability for inclusion. Studies had to be prospective clinical trials that reported to control the conditions, placebo injection and the efficacity of injection of tranexamic acid in the case of SAH or ICH. Furthermore, they had to study mortality during a follow-up period. We followed the Preferred Reporting Items for Systematic Reviews and Meta-Analyses (PRISMA) guidelines.

### Data extraction

Table 1 summarizes important data. Data collection included as much information as possible, first author's name, year of publication, journal of publication, country of the first author, study design (randomized-controlled-trial or not), type of bleed (SAH or ICH), diagnostic criteria of bleeding (acute headache, meningeal irritation, blood-stained cerebrospinal fluid not due to trauma, angiography, CT-scan), number and name of groups, number of men in each group, number of women in each group, time of mortality reported, duration of treatment, posology of therapy, number of patients in each group, number of dead patients in each group, neurologic status (Botterell classification, Hunt and Hess classification^6^, World Federation of Neurologic Surgeon—WFNS classification^7^, Glasgow Coma Scale^8^, National Institute of Health Stroke Score—NIHSS^9^) and mean age of groups. When the mean age was not described but the population was distributed, we used the following formula to calculate mean age: S[(n_i_ x (min_i _+ max_i_)/2] where n is the number of people, i the occurrence, min the minimal age of the event and max the maximal age of the occurrence. Considering we have an event (dead or not dead) in two different populations (exposed to tranexamic acid or not), we decided to calculate all risk ratio (with 95% interval) for all studies to homogenize results.

**Table 1:** Description of characteristics of included studies.

Study	Country	Type of bleed	Design	Method of diagnosis	Groups	Men (n)	Women (n)	Duration offollow-up	Duration of treatment	Treatment protocol	Clinical score
Chandra 1978	Indonesia	SAH	RCT	Lumbar puncture + angiography	TXA and control	21	18	21 days	3 weeks	IV or Per Mouth1g/4 h	Own
Fodstad 1978	Sweden	SAH	RCT	Lumbar puncture + angiography	TXA and control	23	23	> 3 months	6 weeks	IV 1 g/4 h 1w then 1 g/6 h 4w then 1 g/8 h 1w	Botterell
Fodstad 1981	Sweden	SAH	RCT	Lumbar puncture + angiography	TXA and control	22	19	90 days	5 weeks	IV 1 g/4 h 1w then 1 g/6 h 4w	Botterell
Gelmers 1980	Netherlands	SAH	Non-RCT	Lumbar puncture	TXA and control	26	31	90 days	3 months	IV 1 g/6 h	Botterell
Gibbs 1971	UK	SAH	RCT	Angiography	TXA and control	17	30	2 months	3 weeks	Per Mouth 3 g/day	?
Hillman 1997	Sweden	SAH	RCT	CT-scan	TXA and control	330	175	> 3 months	3 days	IV 1 g bolus then 1 g 2 h after bolus then 1 g/6 h	Hunt and Hess
Kaste 1979	Finland	SAH	RCT	Lumbar puncture	TXA and control	30	34	21 days	21 days	IV 1 g/4 h	Botterell
Maurice-Williams 1978	UK	SAH	RCT	Angiography	TXA and control	?	?	42 days	42 days	IV 1 g/4 h 1w then 1.5 g/6 h per mouth	Botterell
Meretoja 2020	Australia	ICH	RCT	CT-scan	TXA and control	52	48	90 days	?	IV 1 g bolus then 1 g/8 h	Glasgow/NIHSS
Post 2021	Netherlands	SAH	RCT	CT-scan	TXA and control	312	644	30 days	1 day	IV 1 g bolus then 1 g/8 h	WFNS
Sprigg 2014	UK	ICH	RCT	CT-scan	TXA and control	14	10	90 days	?	IV 1 g bolus then 1 g/8 h	Glasgow/NIHSS
Sprigg 2018	UK	ICH	RCT	CT-scan	TXA and control	1301	1024	90 days	?	IV 1 g bolus then 1 g/8 h	Glasgow/NIHSS
Tsementzis 1990	UK	SAH	RCT	Angiography	TXA and control	46	54	28 days	4 weeks	IV 1.5 g/4 h	Botterell
Van Rossum 1977	Netherlands	SAH	RCT	Lumbar puncture	TXA and control	?	?	90 days	10 days	IV 1 g/6 h	?
Vermeulen 1984	Netherlands	SAH	RCT	Angiography	TXA and control	189	290	90 days	4 weeks	IV 1 g/4 h 1w then 1 g/6 h 3w	Hunt and Hess

### Quality of articles

We used the Scottish Intercollegiate Guidelines Network (SIGN) criteria to check the quality of randomized clinical trials comprising 10 items^10^. Three authors (JBBM, CC, and NP) evaluated the methodological quality of the studies. Items were assessed for leading causes of bias. We calculated an overall quality score by quoting each item (yes—1 point; no, can't say or not applicable—0 point). Each study had a score between 0 and 10.

### Data analysis

Statistical analysis was conducted using Stata software^©^ (v16, StataCorp, College Station, US). Baseline characteristics were summarized for each study sample and reported as means ± standard deviations and number (%) for continuous and categorical variables, respectively. Effect sizes were defined as relative risk. Random effects meta-analyses (DerSimonian and Laird approach) were conducted when data could be pooled. P values less than 0.05 were considered statistically significant. We conducted a meta-analysis on the impact (risk ratio) of TXA on mortality in NTIB. Risk ratio < 1 with a 95%CI not containing 1 reflected a decreased risk of mortality with TXA, and a risk ratio > 1 denoted an increased risk of mortality. We assessed statistical heterogeneity between results by examining forest plots, confidence intervals, and using I^2^, which is the most common metric for measuring the magnitude of between-study heterogeneity and is easily interpretable. I^2^ is a percentage and is typically considered as low if heterogeneity scores < 25%, modest if between 25 and 50%, and high if > 50%^11^. For rigor, we used a funnel plot of the meta-analysis to search for potential publication bias. We used visual inspection of funnel plot asymmetry to assess publication bias sensitivity analysis, excluding studies not evenly distributed around the funnel base. We secondly used Egger’s test to determine other biases. To verify the strength of the results', a further meta-analysis was then conducted, excluding studies that were not evenly distributed outside of the metafunnel. We also further computed another sensitivity meta-analysis by considering only randomized controlled trials. Furthermore, we proposed stratification by the type of bleeding (SAH or ICH) and meta-regressions to study the relationship between the use of TXA, and age, sex, year of publication, duration of treatment, or severity of the intracranial bleeding. We expressed the results of the meta-regression as regression coefficients at 95% CI.

## Results

**Figure 1:**
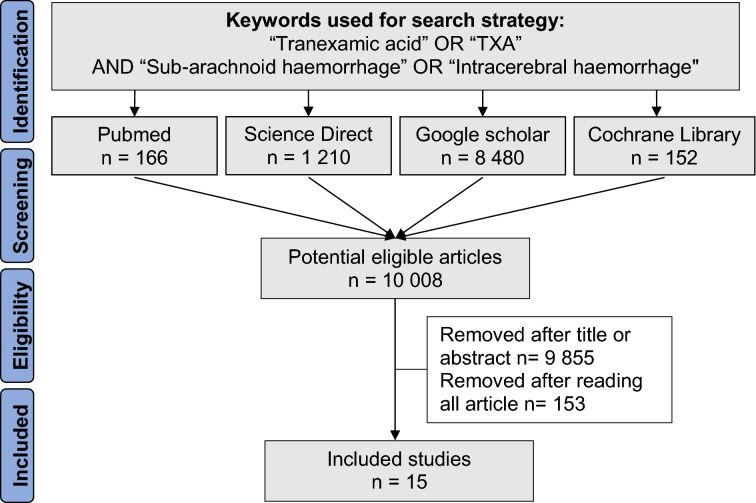
Search strategy.

**Figure 2:**
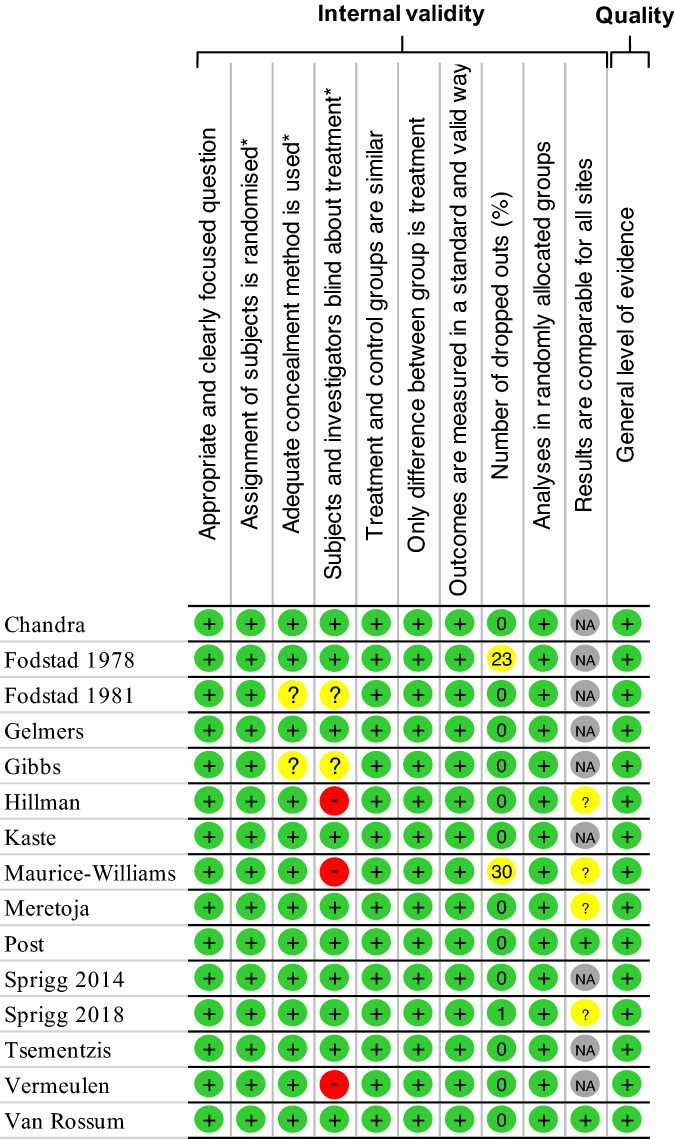
Quality of articles.

In our initial search, we found a total of 10,008 articles. Removal of duplicates and use of the selection criteria reduced the number of articles to 15^12–26^ (Fig. 1). All articles were written in English. Using the SIGN criteria, all the 15 included studies had a score of > 7/10, and were considered high-quality (Fig. 2). All studies had ethical approval. The date of publication was between 1971^16^ and January 2021^20^.

### Patient characteristics

4883 patients were included in our analysis: 2455 in the TXA group and 2428 in the control group. The sample size ranged from 8^22^ to 2325^23^. All participants were adults > 18 years old with a NTIB. Age was reported in thirteen studies, either as mean^14,16,20–23,25,26^ or age class^12,13,15,18,24^. Most patients (> 90%) were under 70 years old. Gender was reported in 13 studies^12–18,20–25^. The mean percentage of men was 51% (95%CI 50 to 53%), ranging from 29.4^27^ to 70%^20^.

### Study designs of included articles

All studies outlined either TXA or a placebo, except five^14,15,17,19,27^. Thirteen studies were randomized controlled trials^12–14,17–20,22–26^ and one was a non-randomized controlled trial^15,27^. The duration of follow-up ranged from 21 days^12^ to > 3 months^13,17^. Eight studies were monocentric^12–15,18,22,24^ and seven were multi-centric^17,19,20,23,25–27^.

### Diagnosis and severity of bleeding

The diagnosis of intracranial bleeding contained clinical criteria in all studies. Validation was performed following results of a lumbar puncture only in three studies^15,18,26^, after lumbar puncture and cerebral angiography in three studies^12–14^, only angiography in four studies^16,19,24,25^, CT-scan in four studies^17,20,22,23^ and after CT-scan or results of lumbar puncture in one study^27^. The severity of NTIB was evaluated using the Botterell classification in six studies^13–15,18,19,24^, the Hunt and Hess scale in two studies^17,25^, the WFNS in one study^27^, the combination of GCS and NIHSS in three studies^20,22,23^, an individual clinical score in one study^12^, and was not evaluated in two studies^16,26^.

### TXA protocol

TXA or placebo was injected intravenously in all studies except one that administered it by mouth three times a day^16^. TXA injection frequency ranged between 1 g every 8 h^20,22,23^ to 1.5 g every 4 h^24^. Placebo was injected at the same frequency as TXA and was NaCl 0.9% in all studies except one which used Epsilon Amino Caproic Acid (EACA)^16^. All studies specified that patients of both groups received the same regime of good nursing and medical care with attention to antihypertensive therapy and adequate analgesia and sedation. Only four studies^13,18,19,24^ defined the cause of death between "total death" and "death secondary to rebleeding".

### Meta-analysis on the effect of TXA mortality on all bleeding

**Figure 3:**
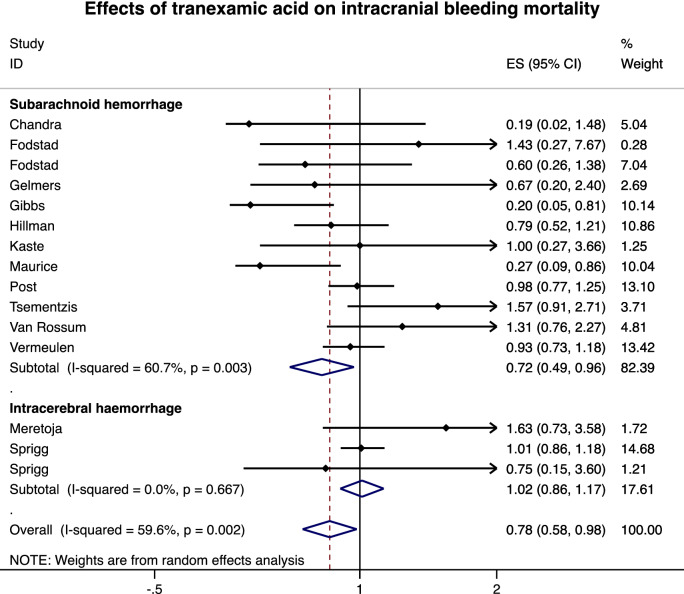
Effects of tranexamic acid on ICH mortality. From Stata software^©^ (v16, StataCorp, College Station, US).

**Figure 4:**
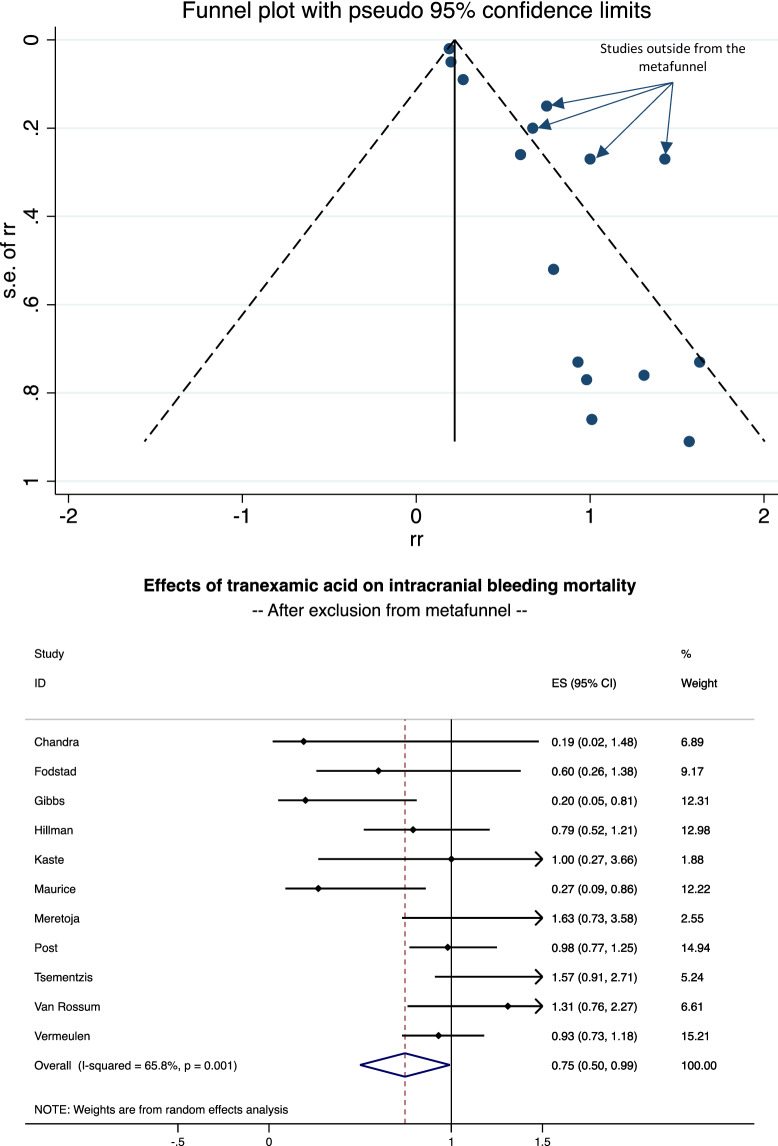
Effects of tranexamic acid on ICH mortality After exclusion from metafunnel. From Stata software^©^ (v16, StataCorp, College Station, US).

**Figure 5:**
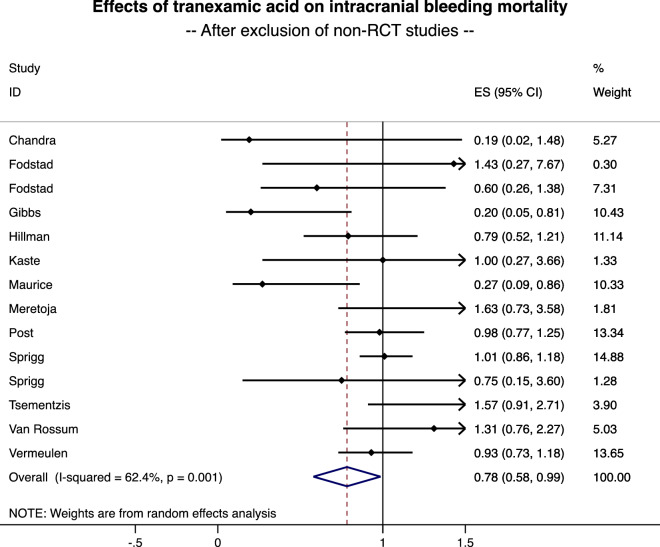
Effects of tranexamic acid on ICH mortality After exclusion of non-RCT studies**.** From Stata software^©^ (v16, StataCorp, College Station, US).

Among the 4883 patients (2455 in the TXA group and 2428 in the control group), 1110 died (22.7%) during the follow-up—546 in the TXA group (22.2%) and 564 (23.2%) in the control group. The meta-analysis of 15 studies demonstrated a 22% decrease in mortality for patients treated by TXA (RR = 0.78, 95%CI 0.58 to 0.98, p < 0.001) (Fig. 3). Heterogeneity between studies was high (I^2^ = 59.6%). Stratification by type of bleeding demonstrated a significant decrease in mortality in the case of SAH (0.72, 0.49 to 0.96, p < 0.001) but a non-significant impact of TXA in the case of ICH (1.02, 0.86 to 1.17, NS). Meta-regression did not demonstrate an influence of the severity of bleeding, age, sex, duration of treatment or date of publication. Visual asymmetric impression of the funnel plot and Egger’s test (p = 0.532) suggest small publication bias. Sensitivity analyses demonstrated benefits of TXA on mortality, with a 25% decrease in mortality after exclusion of studies outside the metafunnel (RR = 0.75, 95%CI 0.50 to 0.99, p < 0.001) (Fig. 4), and with a 22% decrease in mortality when considering only RCT (0.78, 0.58 to 0.99, p < 0.001) (Fig. 5).

## Discussion

To our knowledge, this is the first systematic review and meta-analysis studying the impact of TXA on mortality among patients suffering from NTIB (SAH and ICH). Among the 15 included studies, ten had an RR < 1 which was non-significant. Our meta-analysis demonstrated a possible significant benefit of TXA on mortality in NTIB patients. Furthermore, we failed to show a significant impact of age, sex, date of publication, duration of treatment or severity of initial bleeding on mortality after meta-regression. This result suggests that TXA can be largely prescribed to treat non-traumatic intracranial bleeding. We chose to use the “random-effect model”, which is a more realistic assumption of results. Indeed, patients characteristics, treatment administration and outcomes are not the same in all studies (and relate to patient’s care)^28^.

### The challenge of TXA in SAH and ICH treatment

Spontaneous—non-traumatic—intracranial bleeding—i.e., non-traumatic—is composed of two main entities with different anatomic and pathophysiologic causes. The first one is spontaneous subarachnoid hemorrhage (SAH). It is a severe stroke type mainly caused by the rupture of an aneurysm in the subarachnoid space. The leading cause of premature death in SAH is rebleeding^21^. Rebleeding must be prevented as early as possible by endovascular or neurosurgical obliteration of the ruptured aneurysm. The challenge of TXA treatment is to give time for the obliteration of the aneurysm. The second type of hemorrahage is intracerebral hemorrhage (ICH). Unfortunately, this type is mainly caused by the rupture of very-small vessels in the brain parenchyma. Therefore, vessels responsible for an ICH are not accessible to endovascular or neurosurgical obliteration. The only treatment is lowering blood pressure if needed^23^. Monitoring the condition is still a challenge in neurocritical care^29,30^. In this context, TXA has good potential to decrease mortality in ICH. If our meta-analysis demonstrated evidence-based benefits of TXA in SAH, the benefits in ICH are yet to be outlined and meaningful conclusions may suffer from the low number of studies (12 studies in SAH and three studies in ICH).

### Evolution of care over the last 40 years

The studies included in our review ranged in publication dates from 1971 to 2020. Over the last 40 years, the diagnosis of intracranial bleeding has changed, mainly because of the development of brain computed tomography technology—invented in 1972 and progressively installed in each radiology department with access for emergency patients during the late 1990s and early 2000. Consecutively, angiography, which was the gold standard for SAH and aneurysm diagnosis, has gradually been discarded. Lumbar puncture is limited to cases with a severe headache and clear CT-scan^31^. Care standards for patients suffering from NTIB have also changed over the last 40 years. Prior to the 1990s, aneurysms' late treatment led researchers to develop antifibrinolytics therapies such as aminocaproic acid and tranexamic acid to prevent rebleeding. However, a 2003 Cochrane Review demonstrated an increase in cerebral ischemic events after using antifibrinolytics drugs and recommended not to use antifibrinolytic medicines to treat patients with SAH^32^. Those results seem to reduce the impact of antifibrinolytic therapies because of an increase cerebral ischemia and poor neurological outcomes. However, another meta-analysis^33^ discussed the impact of antifibrinolytic therapies in cerebral ischemia after a subarachnoid hemorrhage. Indeed, antifibrinolytic therapies were administrated during long periods before the years 2000s (up to 3 months^15^) and were not associated with neuroprotective medications such as calcium channel blockers, and used no associated hypervolemic therapies to prevent ischemic insult. They concluded that short-term use of antifibrinolytic therapy (before aneurysm exclusion, always less than 3 days) associated with calcium channel blocker and hypervolemic therapies in the management of aneurysmal SAH diminishes rebleeding rate, and does not augment the risk of cerebral infarction. Since the late 1990s, it is now highly recommended to surgically repair aneurysms as soon as possible. A recent meta-analysis proved the benefit of early and mini-invasive procedures for supra-tentorial spontaneous ICH^34^. The development of intensive care units specialized in neurology with continuous monitoring of intracranial pressure, use of calcium channel blockers to protect the neurological status, and decrease the risk of delayed ischemic stroke have greatly improved SAH's care. Recent developments in endovascular coiling for cerebral aneurysms will probably further reduce post-operative complications. This will also allow elderly patients who are currently deprived of open neurosurgery to benefit from this technique. This will probably become the gold standard in the next decade.

### Other indications of tranexamic acid

When TXA was discovered in the 1960s in Japan, its discoverers hoped it would have a positive effect on postpartum hemorrhage, at a time when dying in childbirth was common. The WOMAN trial^35^, published in 2017, found a protective effect of TXA on post-partum hemorrhage for over 20,000 women from 193 hospitals in 21 countries. This obstetrical indication was progressively extended to heavy menstrual bleeding. Cochrane researchers^36^ recently proved a positive effect of TXA on heavy menstrual bleeding compared to placebo (− 53.2 mL per cycle; 95CI − 62.7 to − 43.7), non-steroidal anti-inflammatory drugs, oral luteal progestogens, ethamsylate of herbal remedies. However, it may be less effective than the levonorgestrel intrauterine system. In 2011, the CRASH-2 trial^5^ found a statistical benefit of TXA within 3 h following injury on patients with traumatic extracranial bleeding (RR 0.85, 95CI 0.76 to 0.96). This finding resulted in including TXA in the WHO model list of essential medicines for traumatic bleeding. In 2019, the CRASH-3 trial^37^ assessed a slight—although not statistically significant—effect on patients with traumatic brain injury when injected within 3 h (RR 0.94, 95CI 0.86 to 1.02). A sub-group analysis found a benefit on patients suffering from mild to moderate brain injury (RR 0.78, 95CI 0.64 to 0.95). However, in 2020, a meta-analysis of nine randomized controlled trials did not show any benefit of TXA on traumatic brain injury (RR 0.95, 95CI 0.88 to 1.02). This study concluded that TXA might decrease hematoma expansion on subsequent imaging and not increase the risk of adverse events^38^. However, a further study found that prehospital tranexamic acid administration was associated with increased mortality in patients with isolated severe TBI^39^. The HALT-IT randomized, double-blind, placebo-controlled trial^40^, also published in 2020, did not benefit patients suffering from acute gastrointestinal bleeding on 5 days mortality (RR 0.99, 95CI 0.82 to 1.18). TXA is also used in emergent hemoptysis or epistaxis and in planned orthopedic or cardiac surgery, which were not studied because of their weak mortality.

### Limitations

Our study has some limitations. Firstly, we decided to pool both SAH and ICH although those two pathologies have different populations, etiology and some difference in standard of care. To decrease the impact of this choice, we stratified our meta-analysis by type of bleeding (SAH versus ICH). Also, the main interventions for the care of intracranial bleeding are surgery and critical care. This is an important bias for the generalization of our results. Indeed, a hospital without neurosurgeons or interventional radiologists and critical care physicians cannot focus on the care associated with TXA in isolation. However, all patients included in all studies had the same access to neurosurgeons, interventional radiologists, and critical care physicians. Secondly, the difference in diagnosis and care between the 1970s and the 2020s induced a bias. However, no difference was found in mortality based on publication date. Thirdly, all trials' primary outcomes, treatment protocols, and duration of follow-ups were not identical. We also included both RCT and non-RCT trial which can decrease the power of generalization of our results. Fourthly, the duration of follow-up is heterogenic from 21 days through to 3 months duration between all studies. This could be an important bias for generalization. Fifthly, the inherent limitations of meta-analysis, such as publication bias, cannot be ignored. Lastly, the severity classifications were not similar. However, classification grades have been clustered in the meta-regression that did not find any effect. We did not study the adverse effect of TXA because many studies recently published proved its safety under many conditions, such as melasma in 2017^41^, obstetric in 2018^42^, orthopedic surgery in 2018^43^, craniomaxillofacial surgery in 2016^44^, and pre-hospital traumatic hemorrhagic shock in 2016^44^.

## Conclusion

Tranexamic acid, a lysin analog, appears to be a therapeutic option to reduce non-traumatic intracranial bleeding mortality, particularly in patients with SAH. Its effects on mortality on ICH are yet unclear and require further investigation.

## Data Availability

All data are available on the different articles.
